# The Future of the Multidisciplinary Clinic

**DOI:** 10.1100/tsw.2007.254

**Published:** 2007-11-12

**Authors:** Timothy J. Brei

**Affiliations:** Indiana University School of Medicine, Section of Developmental Pediatrics, James Whitcomb Riley Hospital for Children, 702 Barnhill Drive, Indianapolis, IN 46202, USA

**Keywords:** spina bifida, myelominingocele, multidisciplinary clinic, delivery of health care, health services

## Abstract

The multidisciplinary clinic is the accepted model for health care delivery related to spina bifida. This article focuses on the factors affecting multidisciplinary care delivery and future challenges for multidisciplinary programs.

Myelomeningocele, which results from abnormal formation of the neural tube, is one of the most complex birth defects compatible with life. It affects multiple body systems with resultant widespread changes in functions. Individuals with myelomeningocele, more commonly referred to as spina bifida (SB), usually have hydrocephalus; other abnormalities of the nervous system, such as the Chiari II malformation and tethered spinal cord, may also occur. These neurologic impairments of SB lead to varying degrees of paralysis, neurogenic bladder and bowel, and sensory impairments. Additional problems can be seen, including neuropsychological deficits, brainstem dysfunction, strabismus, and seizures. Individuals with SB are at high risk for the development of secondary medical conditions, including ventricular shunt malfunction or infection, orthopedic problems like scoliosis and joint contractures, urinary tract infections and other urologic problems like ureteral reflux, pressure ulcers, and obesity. The occurrence of both primary and secondary problems in SB contributes to impaired mobility and continence**.** Persons with SB may have restricted socialization and mental health problems, including depression[1,2,3]**.** They may also experience reduced academic achievement. These challenges place individuals with SB at risk for problems with independence and underemployment, and often do not allow persons with SB to participate fully in society.

The complexity of the disorder, the need for involvement of multiple disciplines, and the recognition of the challenges of providing health care to children with SB led to the development of multidisciplinary teams and clinics in the 1960s as a mechanism to improve coordination and integration of needed medical services[4,5]. Multidisciplinary clinics have long been advocated by the Spina Bifida Association as the optimal way to provide care for individuals with SB.

The scope of needs adds to the challenges in providing care to persons with SB. Once focused primarily on a core set of medical disciplines, there is increasing recognition of the need for multidisciplinary clinics to help persons and families affected by SB to address mental health, academic, recreation, and health promotion needs in order to improve daily function and societal participation. There is little agreement about what services must/should be offered and how those services should be provided.

Therefore, while there is widespread distribution of multidisciplinary clinics for SB care, the actual structure of clinics, personnel involved, and funding sources to support multidisciplinary clinics vary widely.

Some literature exists regarding the role of multidisciplinary clinics in other disorders and suggests that multidisciplinary clinics might improve quality of care or lead to improved health outcomes[6]. No such study exists related to SB care, although a single study does exist that documents the negative effects on health of individuals with SB when a multidisciplinary clinic was disbanded[7].

Despite a general lack of published evidence supporting their effectiveness, multidisciplinary clinics remain the model by which current and future care related to SB can be assessed. Provision of care in a multidisciplinary setting vs. an individual provider setting has some potential advantages[8]. At their best, multidisciplinary clinics are more convenient for families. They also offer the opportunity for care providers to collaborate more effectively about patients being seen, and enhance the ability to improve care coordination.

Yet, multidisciplinary clinics for SB care face many challenges as health care providers and families work together to improve both the quality of care and the quality of life for individuals with SB. Although inter-related, these can loosely be grouped into clinic models/structure, personnel, and funding. Little research in SB exists that evaluates the relative effectiveness and costs of various models of care, the impact of personnel, or financial aspects of multidisciplinary clinics on health outcomes.

## STRUCTURE

Although most clinics providing care to persons with SB are multidisciplinary, there is no universal agreement as to what disciplines should be included. Most clinics offer care in the medical disciplines of orthopedics, urology, and neurosurgery. Many also have a general pediatrician, developmental pediatrician, or pediatric physiatrist present who serves to provide a more holistic view of the patient; often, but not always, this physician serves as the clinic “medical director”. Most clinics also have nursing, occupational therapy, and physical therapy present during clinic.

However, there is increasing recognition of the need not just to manage medical issues related to SB, but also to promote health and to improve the quality of life. Despite the good medical care received by children with SB, many people with SB moving into adulthood are not living independently[9,10], and/or have reduced employment and social opportunities. Increasing evidence is demonstrating that, in addition to primary medical concerns, a person’s beliefs and attitudes, family cohesion and parenting styles, and the effects of neuropsychological deficits on learning and development of “life skills” play an important role in health outcomes, quality of life, and independence for persons with SB[11,12,13,14]. Families and individuals with SB frequently look to health care professionals in the SB clinics for guidance and assistance. To support a holistic approach, individuals with SB and families often require or benefit from other services, including psychology, neuropsychology, social work, nutrition, orthotics, and family services specialists. These may be available either as part of the clinic structure or on a referral basis.

The World Health Organization’s “International Classification of Functioning, Disability and Health” (ICF) provides a conceptual framework looking at body function/structure, activities and participation, and environmental factors in a holistic fashion. In SB, it can be used to identify factors contributing to secondary conditions, to help develop strategies to reduce or prevent them, and to improve societal participation[15].

Kinsman et al. have worked to develop a conceptual model that incorporates the ICF framework that seeks to move from multidisciplinary care with individually set, discipline-specific goals to inter- or transdisciplinary comprehensive, longitudinal care with team agreement and sharing of person/family-centered goals[16]. Implicit in this model is an increased need for communication among team members and dynamic-evolving processes of care. Developing clinic frameworks and models for care coordination that incorporate ways to address medical and psychosocial concerns, activities, and participation is a significant challenge.

There is also a significant need for SB clinics to address transitions to adulthood and for the development of multidisciplinary programs for adults. Adults with SB have ongoing care needs with higher medical expenditures than adults without SB[17] and are at risk for development of preventable secondary conditions, such as decubitus ulcers, leading to expensive hospitalizations[18]. Yet, many fewer multidisciplinary clinics exist for adults than for children, and services provided in those clinics tend to be less comprehensive, leading to fragmented care. Multidisciplinary models for care exist for some disorders in adulthood, but occur more rarely and seldom require the number of health providers that SB does. Developing coordinated systems of care for adults with SB is an increasing imperative that will require enlistment of hospital/institutional/philanthropic support necessary to provide the necessary infrastructure.

Finally, some families elect not to participate in multidisciplinary clinics. Reasons may be varied, but seeking care outside of a multidisciplinary clinic can lead to more fragmented and less coordinated care. These individuals/families may still benefit from some of the resources/supports of the clinic, so development of flexible systems of care and effective communications between providers becomes more important when this situation exists.

## PERSONNEL

The second major component that affects the future of multidisciplinary clinics relates to personnel. While multidisciplinary clinics may be effective and efficient for individuals and families, from an individual provider perspective, most health care providers can see individuals more efficiently in individual private settings than they can in a multidisciplinary setting where multiple providers are trying to see the same children in a defined span of time. Development and maintenance of a good multidisciplinary clinic requires cooperation and enthusiasm of all health care providers.

In some areas, lack of manpower affects the availability of physician specialists. In the case of adults with SB, many specialists do not feel comfortable with or have had inadequate training in medical care of adults with SB. These issues of physician shortages in many pediatric surgical specialties and graduate medical education will need to be addressed for the ongoing success of multidisciplinary clinics.

There is an increasing utilization of nurse practitioners (NPs) and physician assistants (PAs) throughout health care. NPs and PAs are also being utilized by many physicians who provide care to persons with SB in the multidisciplinary clinics. The impact of using these professionals on health care or outcomes for persons with SB is not clear. No study has looked at quality of health care utilizing NPs or PAs in SB care. A randomized, controlled trial has documented no change in health care outcomes between NPs and physician in a primary care setting[19,20]. In addition, emerging literature is also suggesting that strong physician/NP or PA collaboration helps to optimize care in complex situations or settings, and reduced length of hospital stays and costs[21,22].

## FUNDING

The final challenge facing multidisciplinary clinics is that of the funding of services and the cost of health care. Because of the multiple demands for care required by individuals with SB, multidisciplinary clinics do not break even financially. Most multidisciplinary clinics are dependent on the support of their hospitals or parent organizations for financial support. Multidisciplinary clinics require secretarial/scheduling support, and nursing care and coordination. For every hour of direct patient care provided, 24–60 min of indirect care (such as communicating with schools and community therapists, consulting with specialists, approving expenditures, and writing letters for families) is required[23]. The deployment of physical therapy, occupational therapy, dietary, social work, and other support services are additional expenses incurred. The costs for these services are seldom reimbursable and yet are necessary to the overall functions of a multidisciplinary clinic and support for individuals with SB and their families.

To most hospitals, a multidisciplinary SB clinic is at best revenue neutral and more often viewed as a money loser**.** A recent study examining costs of care for children with special health care needs in a comprehensive primary care, hospital-based clinic demonstrated hospital financial losses[24]. The authors did note that outpatient losses were offset by gains in inpatient services. As private insurance payments and governmental program (primarily Medicaid) reimbursements to hospitals level off or decrease, even hospitals with long-established SB multidisciplinary clinics are looking for ways to cut costs. In some cases, this has resulted in cutting services or disbanding clinics entirely[7]. Even now, some specialists, e.g., plastic surgeons, will not treat individuals with SB if they have Medicaid because of the poor reimbursement rates. The problem of Medicaid reimbursement may be even more acute for adults with SB, many of whom are totally dependent on Medicaid funding for health care.

As these outpatient clinics are viewed as costing hospitals money and resources, convincing hospitals to expend funds to support and maintain ongoing programs or to develop entirely new adult programs is particularly challenging. In order for multidisciplinary clinics to continue, health care professionals and health administrators will need to work together closely to ensure that necessary services in existing programs remain available and funds/resources are identified to develop adult programs. A strategy that encompasses and evaluates both inpatient and outpatient revenues may be important. Another important factor is to include calculations of the medical expenses that would be incurred if the patient does not receive adequate care and is hospitalized with a chronic, preventable condition.

Financial and insurance issues also impact a person's ability to access the services of a multidisciplinary program. Health Maintenance Organizations (HMOs) may restrict access of enrollees to in-network providers who may be less familiar with SB-related care, which can lead to poor outcomes or fragmented care.

Some clinics also receive a significant portion of support from Title V (Maternal and Child Health) funds. In this situation, clinic existence becomes dependent on decisions of state legislators and government agencies. With state budget shortfalls, these clinics face ongoing risks of having to cut services or disband.

In order to address financial concerns, innovative partnerships may need to be created between health providers, philanthropic organizations, individuals with SB and their families, hospitals, corporations, insurance companies, and government agencies.

In summary, the multidisciplinary program remains the model for SB-related health care delivery. Critical to this appears to be the role of care coordination. The existence and effectiveness of multidisciplinary care provision is related to multiple programmatic, personnel, and financial factors**.** Further research is needed that informs each factor. The integrity of the current clinics and the ability to create necessary new programs to address the growing population of adults with SB will require finding solutions to address challenges in all these areas.

## ACKNOWLEDGMENT

The author greatly appreciates the efforts of Dr. Greg Liptak for his editorial comments and review of the manuscript.
